# Supplementary figures and images for: Latching dynamics as a basis for short-term recall
Source: PLoS Comput Biol. 2021 Sep 15;17(9):e1008809. doi: 10.1371/journal.pcbi.1008809 (PMC8476040; doi:10.1371/journal.pcbi.1008809)

(a)

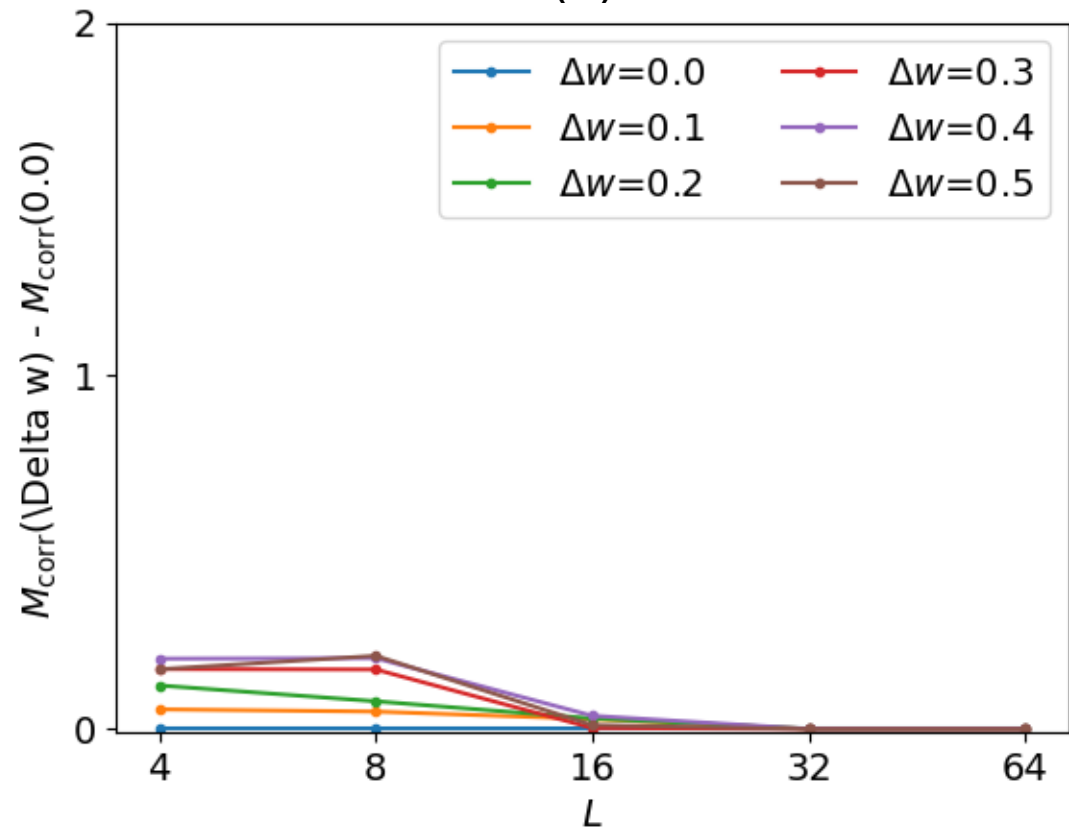

(b)

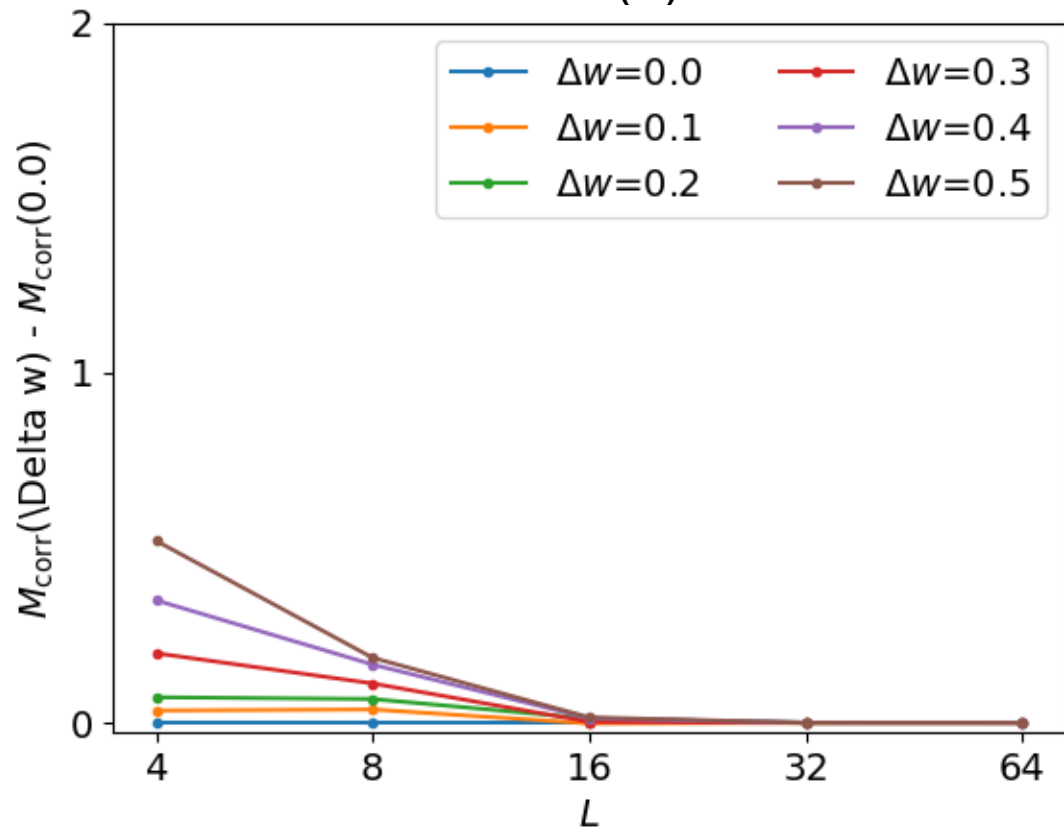

Supplement: S1 Fig — The abscissa is the number of items in STM, L, in a log scale. The ordinate is ΔMcorr ≡ Mcorr(Δw) − Mcorr(0), where Mcorr is the number of recalled STM items until the network either repeats an already-visited item or (mistakenly) retrieves one of the LTM items. Left: w = 1.0, right: w = 1.1. (PDF) [file pcbi.1008809.s001.pdf]

(a)

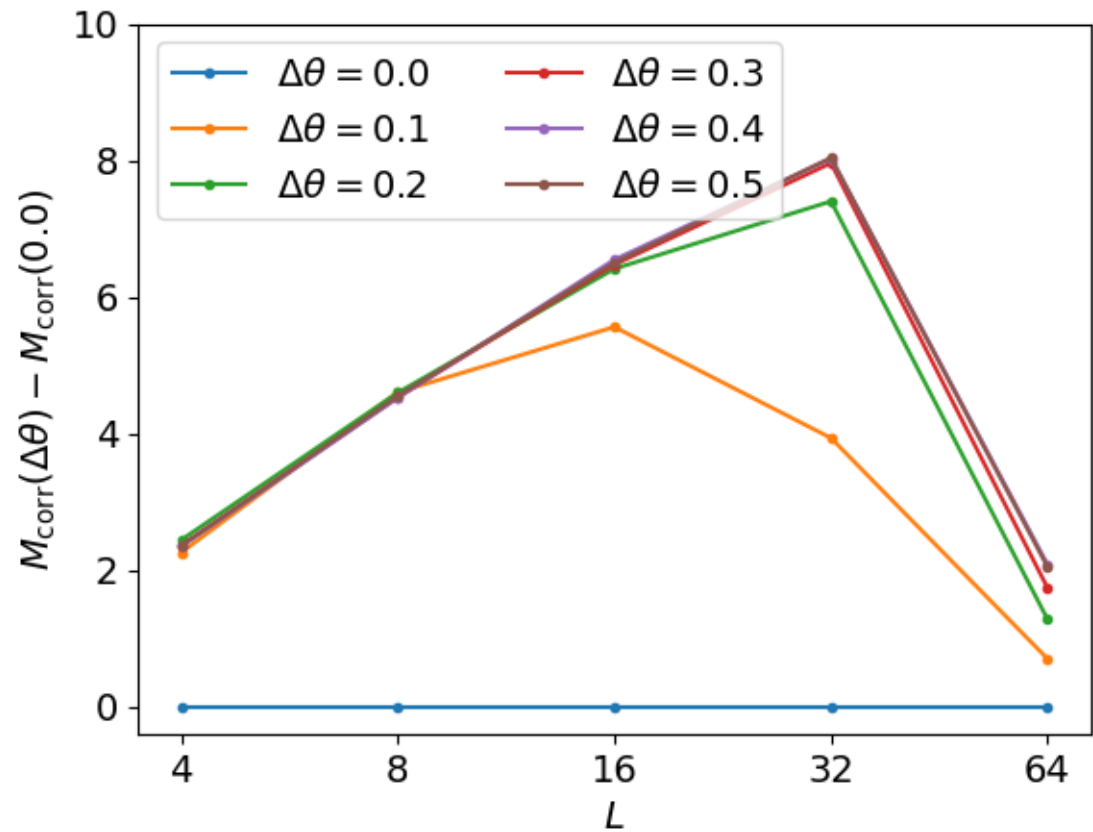

(b)

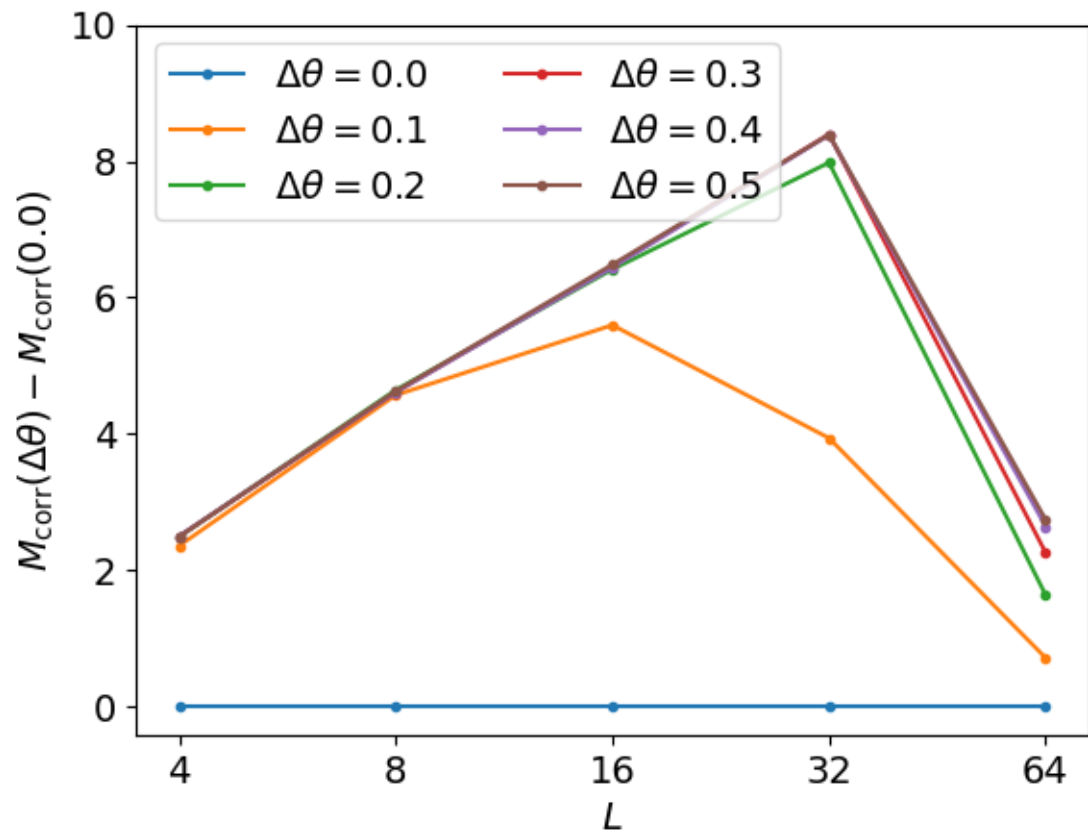

Supplement: S2 Fig — Details as in S1 Fig. (PDF) [file pcbi.1008809.s002.pdf]

(a)

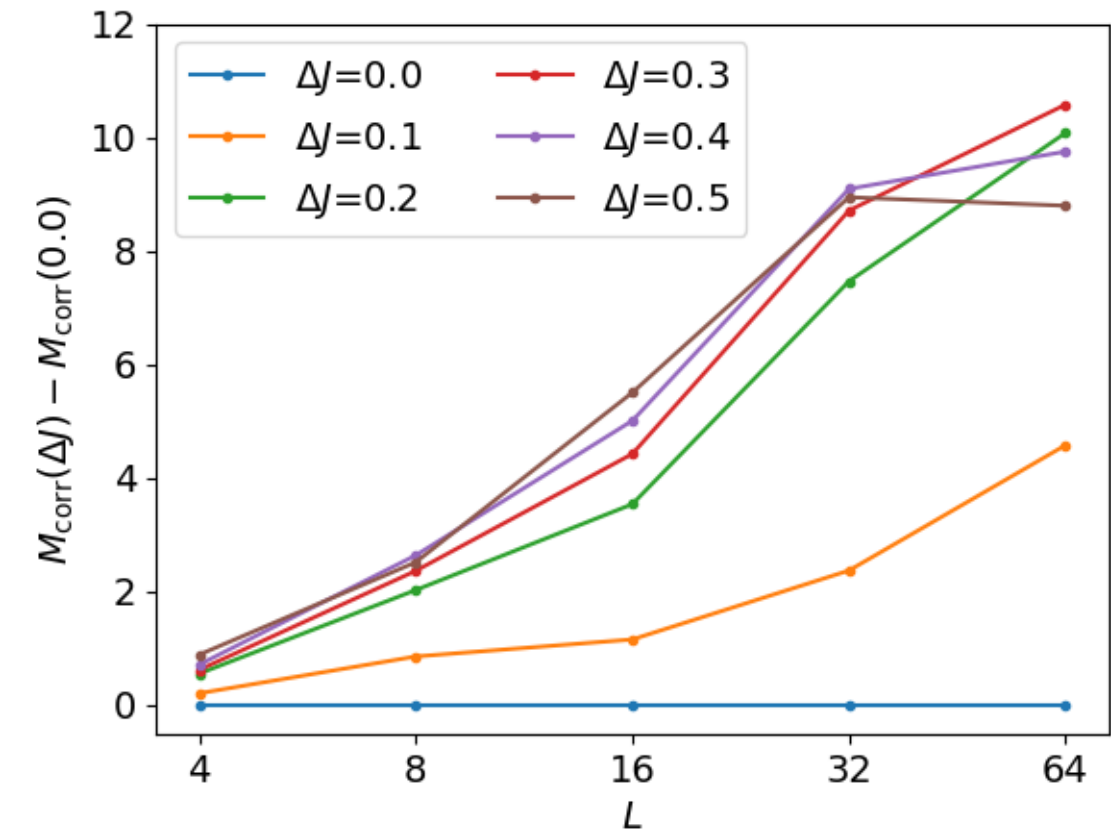

(b)

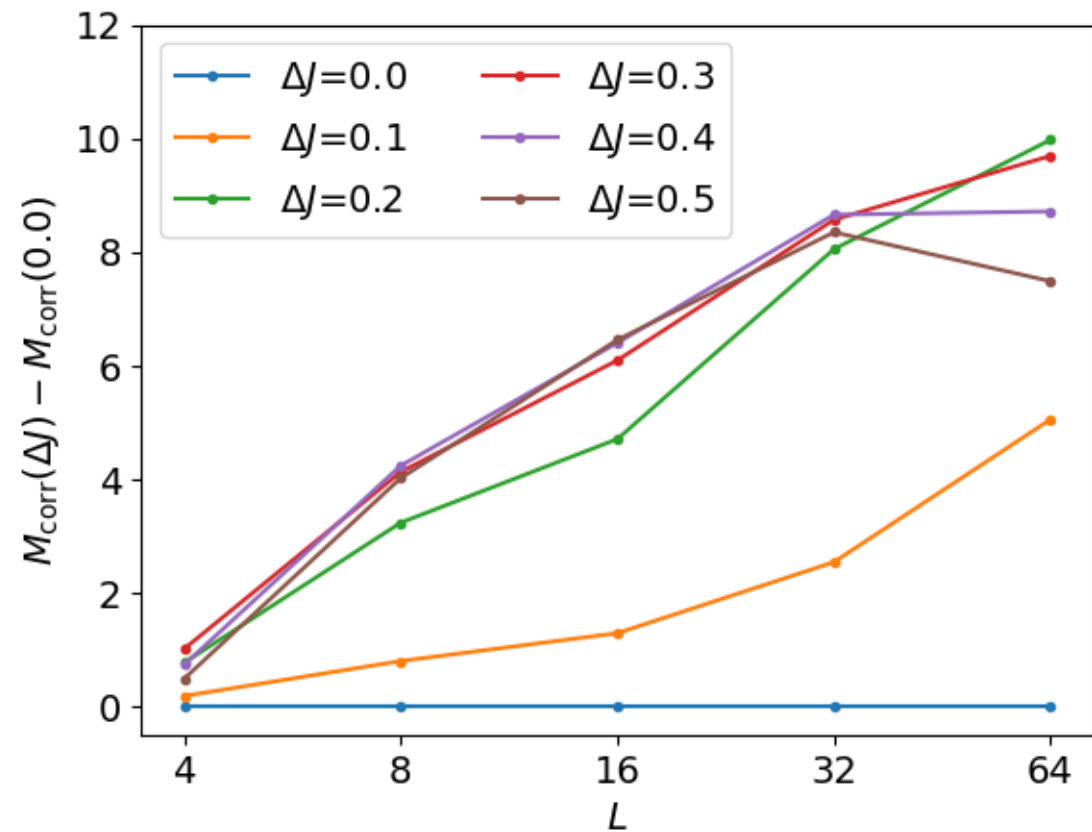

Supplement: S3 Fig — Details as in S1 Fig. (PDF) [file pcbi.1008809.s003.pdf]

(a)

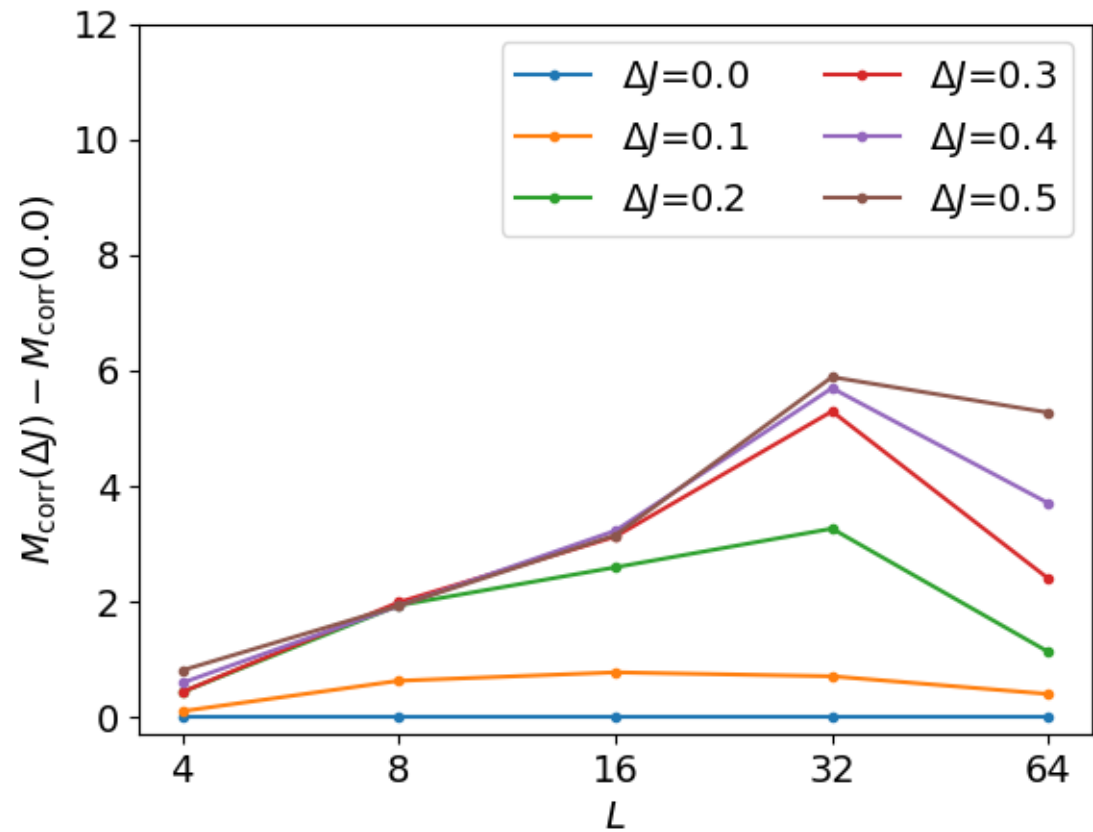

(b)

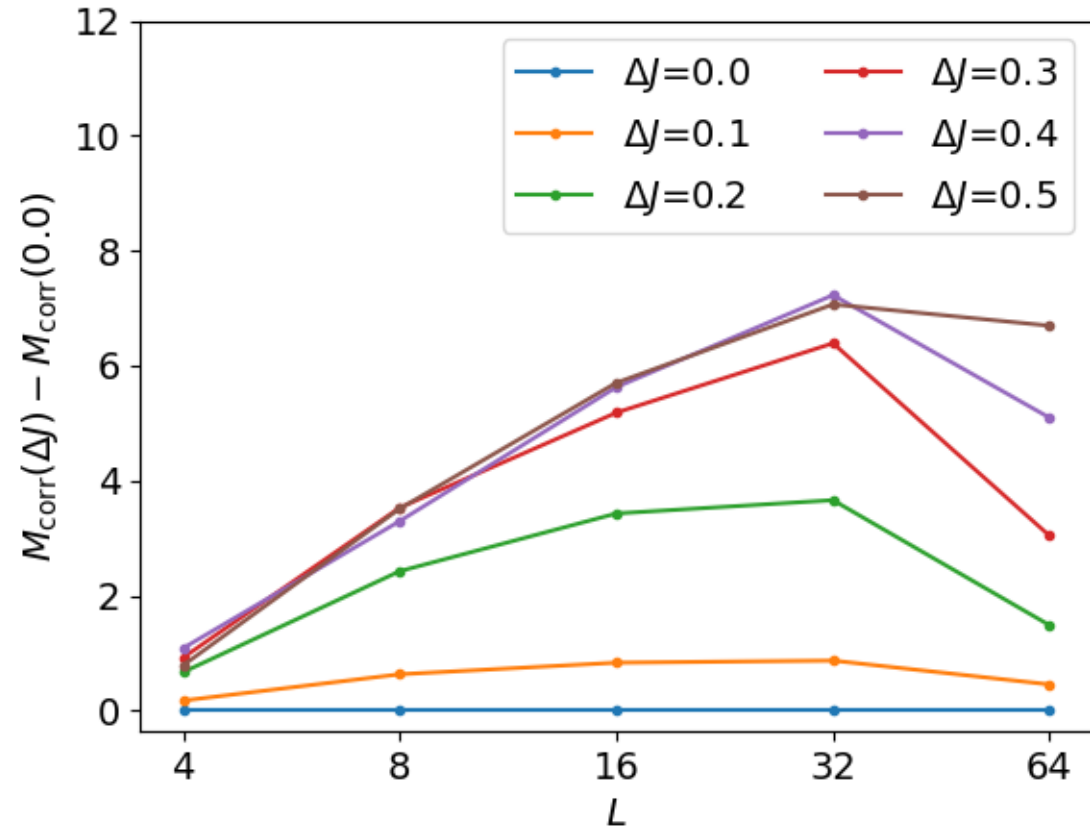

Supplement: S4 Fig — Details as in S1 Fig. (PDF) [file pcbi.1008809.s004.pdf]

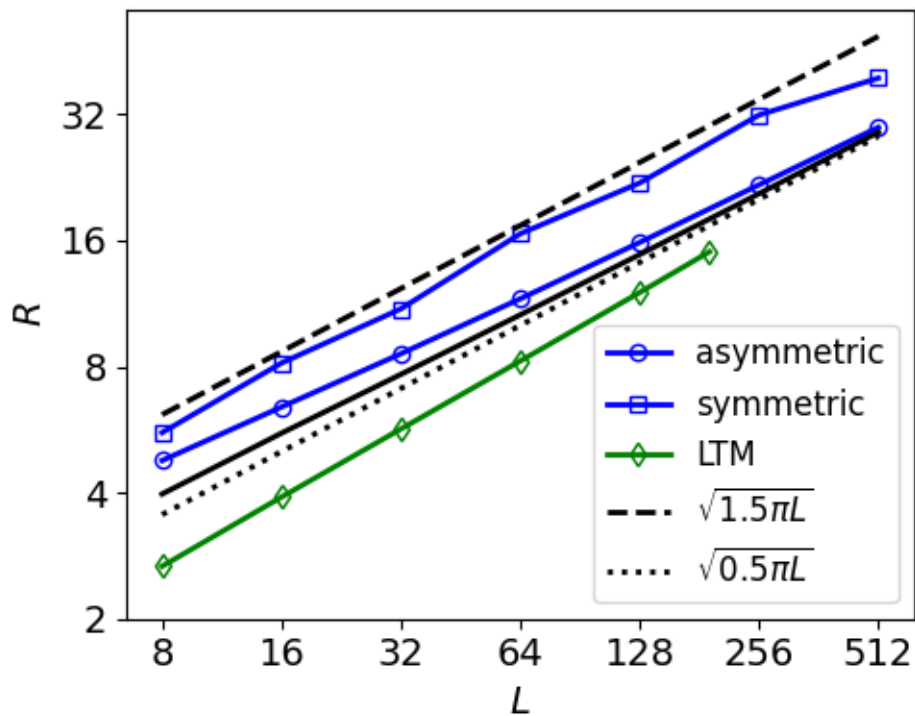

Supplement: S5 Fig — This quantity (R), which is the number of visited STM items until the search process enters a loop, is well-defined only in the case of symmetric similarity matrix. In other cases the quantity R is ill-defined; a closed loop is hardly ever observed in search process, so we compute, instead, Mi1, which is the number of visited STM items until the network revisits one of the already-visited items, as a surrogate for R. The blue curve with squares is R(L) obtained from simulating SAM++ model with random symmetric similarity matrices (1000 simulations). The blue curve with circles is R(L) obtained from simulating SAM++ model with random non-symmetric similarity matrices (10000 simulations). In both cases elements of similarity matrices are drawn from a uniform distribution between 0 and 1. In the latter case, the degree of symmetry is 0.5 on average. The green line with diamonds is R(L) obtained from simulations of the Potts model without short-term boost in the intermediate inhibition regime (γA = 0.5, w = 1.4). We randomly pick L out of p = 200 patterns and treat them as if they were STM items. The solid black line is from the numerical evaluation of Eq (1) in S1 Appendix, which is derived from an equal-probability assumption. All lines shown here have a slope of approximately 0.5. (PDF) [file pcbi.1008809.s005.pdf]

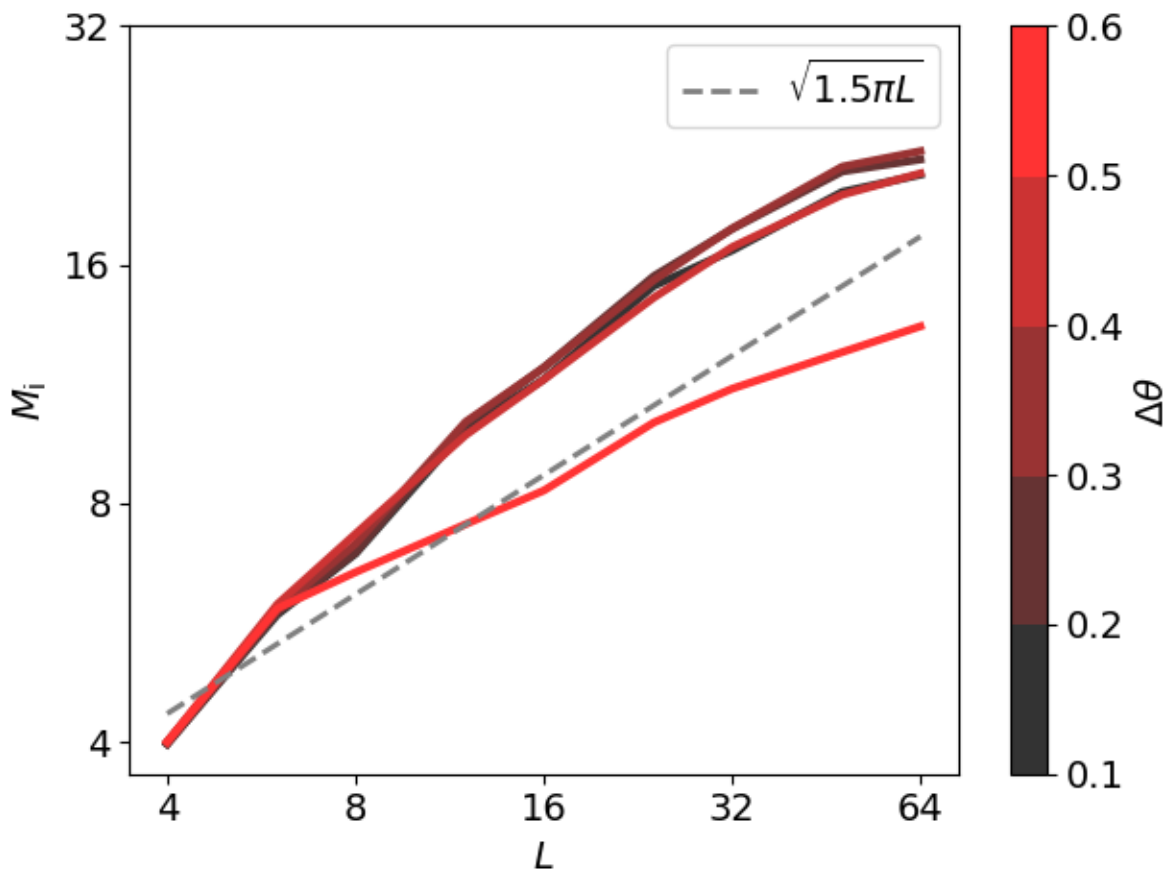

Supplement: S6 Fig — Mi(L) is plotted for several values of Δθ from simulating Model 2. Mi is the number of recalled STM items until one of them is repeated twice. (PDF) [file pcbi.1008809.s006.pdf]

(a)

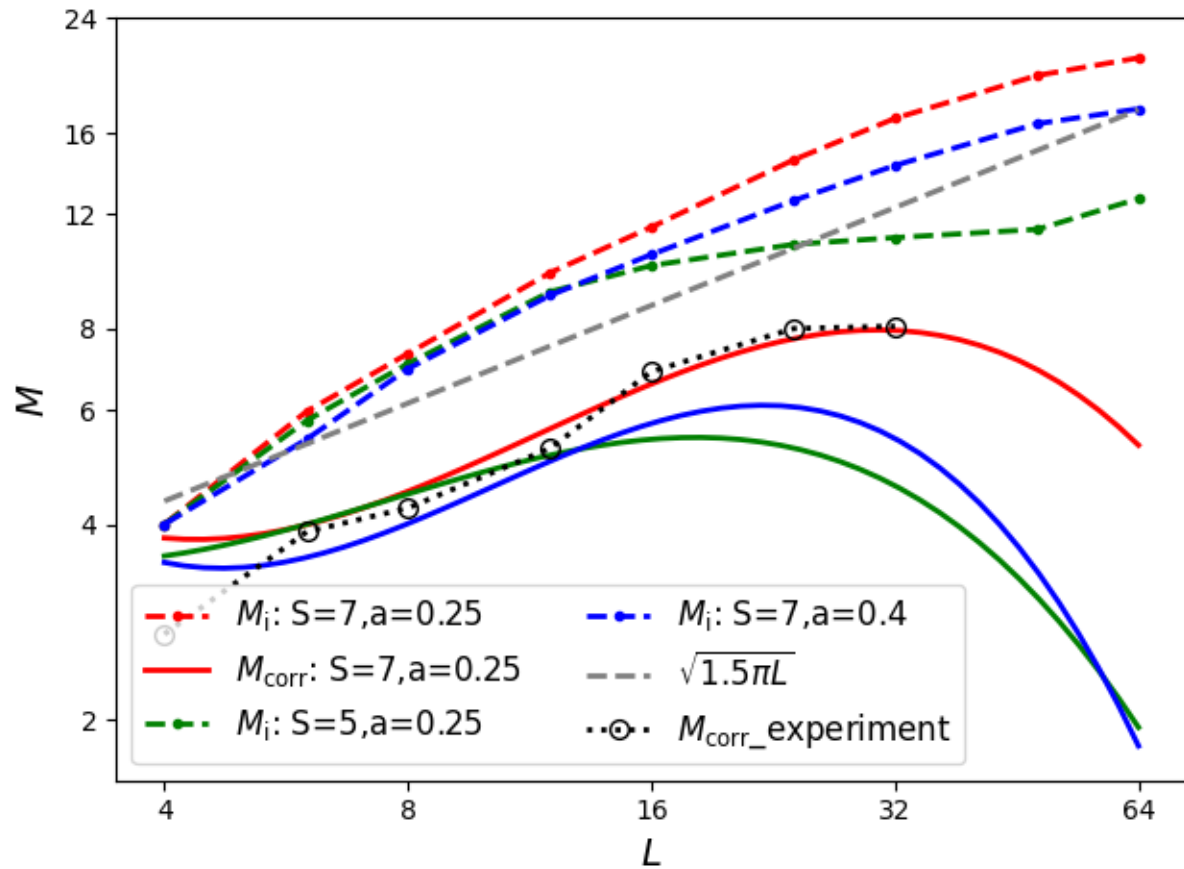

(b)

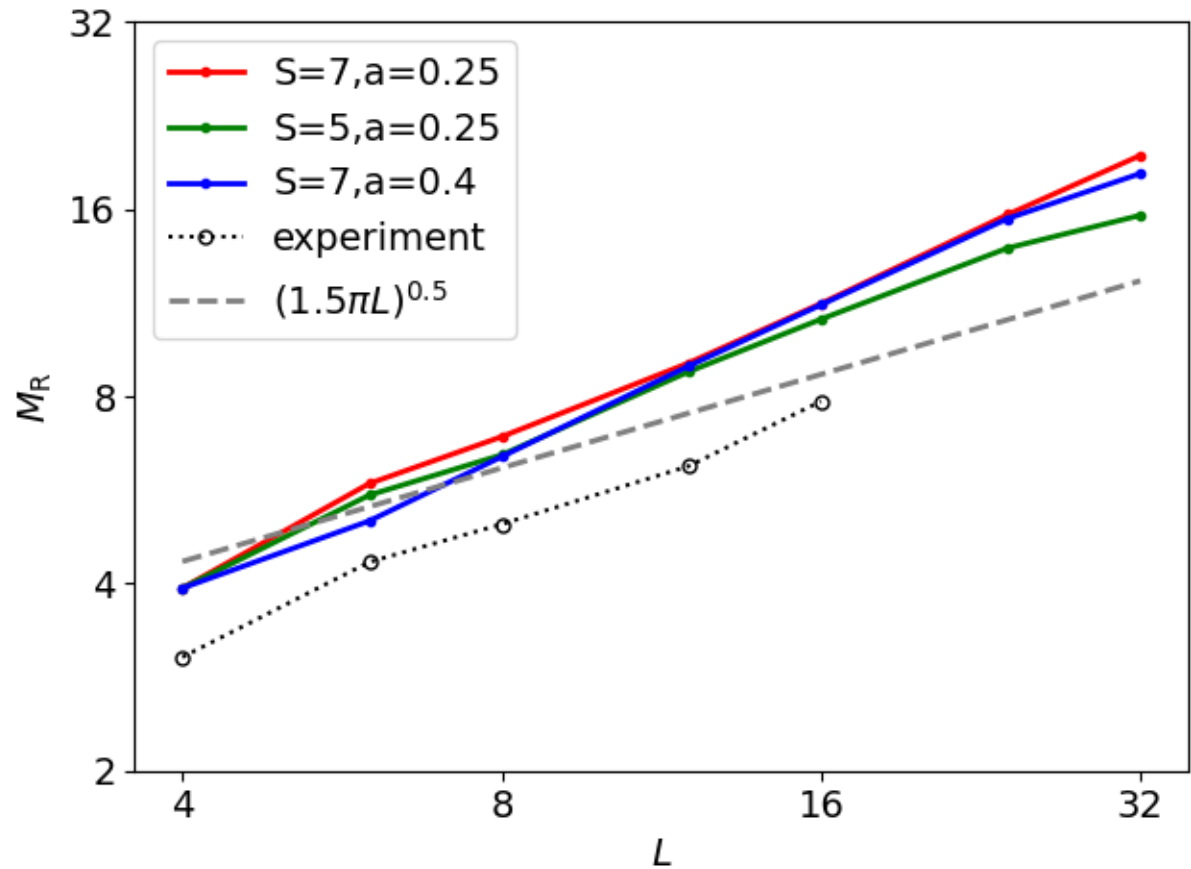

Supplement: S7 Fig — These quantities remain qualitatively the same with respect to changes in S and a, as long as latching dynamics are stably maintained under these changes. Mcorr is the number of recalled STM items until the network either revisits one of the already-recalled STM items or visits one of the LTM items, but within a given number of latches − 2(L − h(t|L)), where h(t|L) is the number of correctly recalled STM items up to that point in time. MR is the number of correctly retrieved STM items within a given number of consecutive latches set as 2(L − h(t|L)), ignoring errors and repetitions. (PDF) [file pcbi.1008809.s007.pdf]

(a)

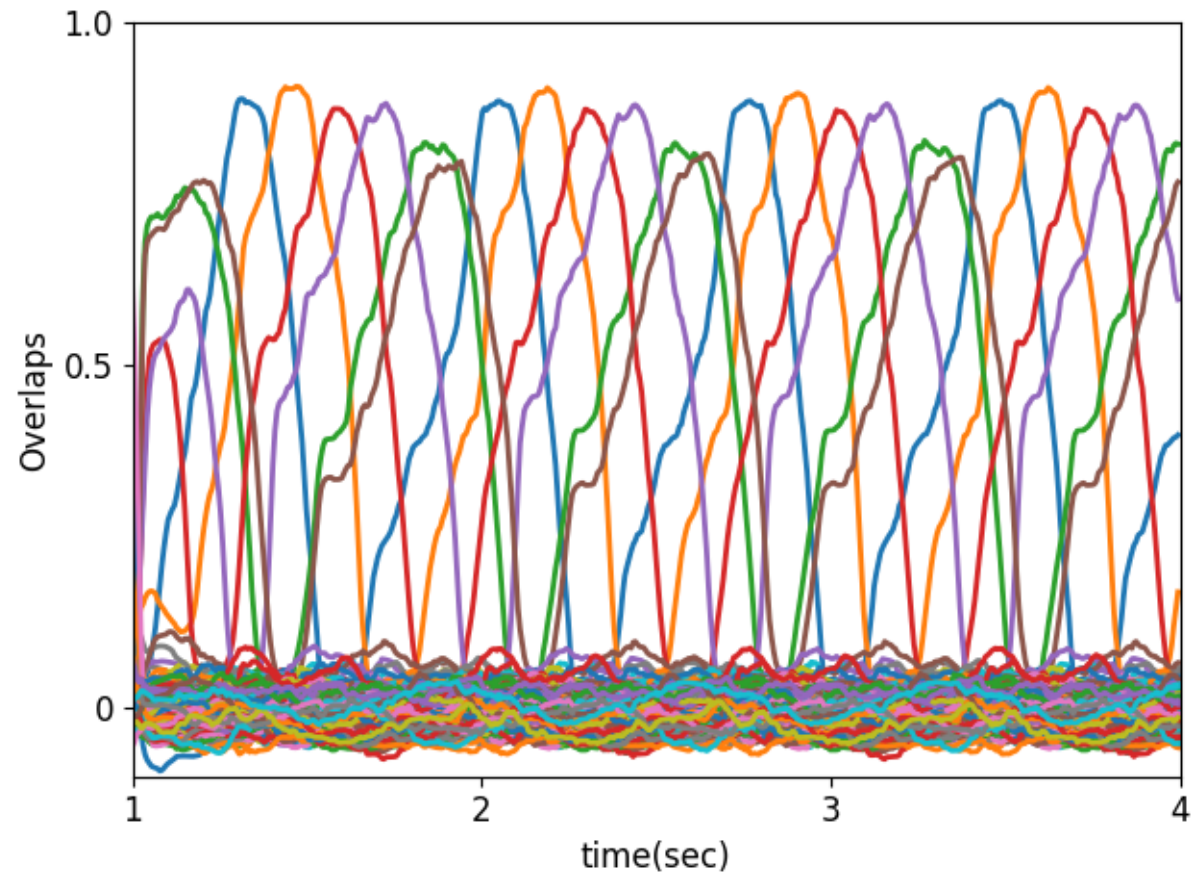

(b)

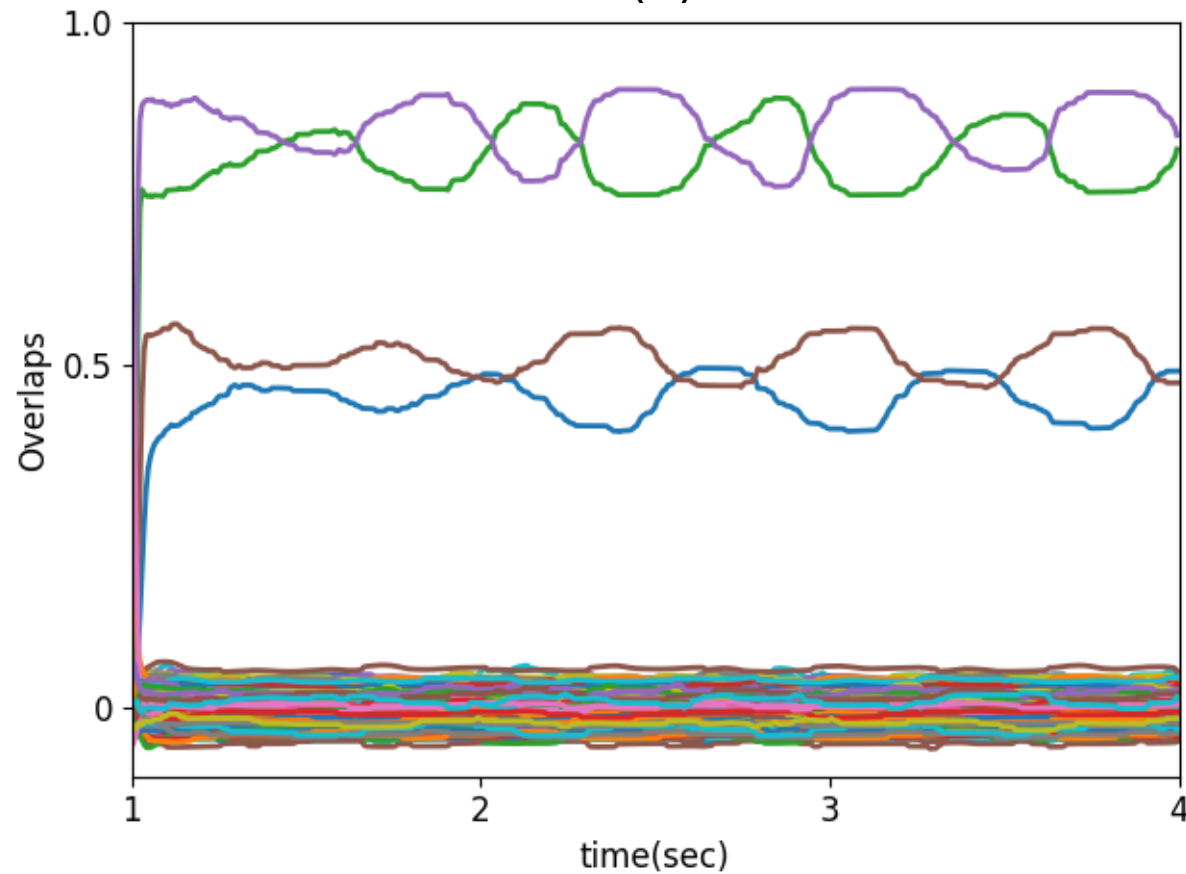

Supplement: S8 Fig — In serial recall by the Potts model, too high values of λ, relative strength of heteroassociative connections to the autoassociative ones, lead to faltering latching dynamics. Two example sequences are shown, for the same parameter values: ω = 1.0, γA = 0.5, Δθ = 0.1, λ = 0.05. Each colour corresponds to a different pattern. The proportion of simulation in which latching completely fails, as in the right panel, increases with λ. (PDF) [file pcbi.1008809.s008.pdf]

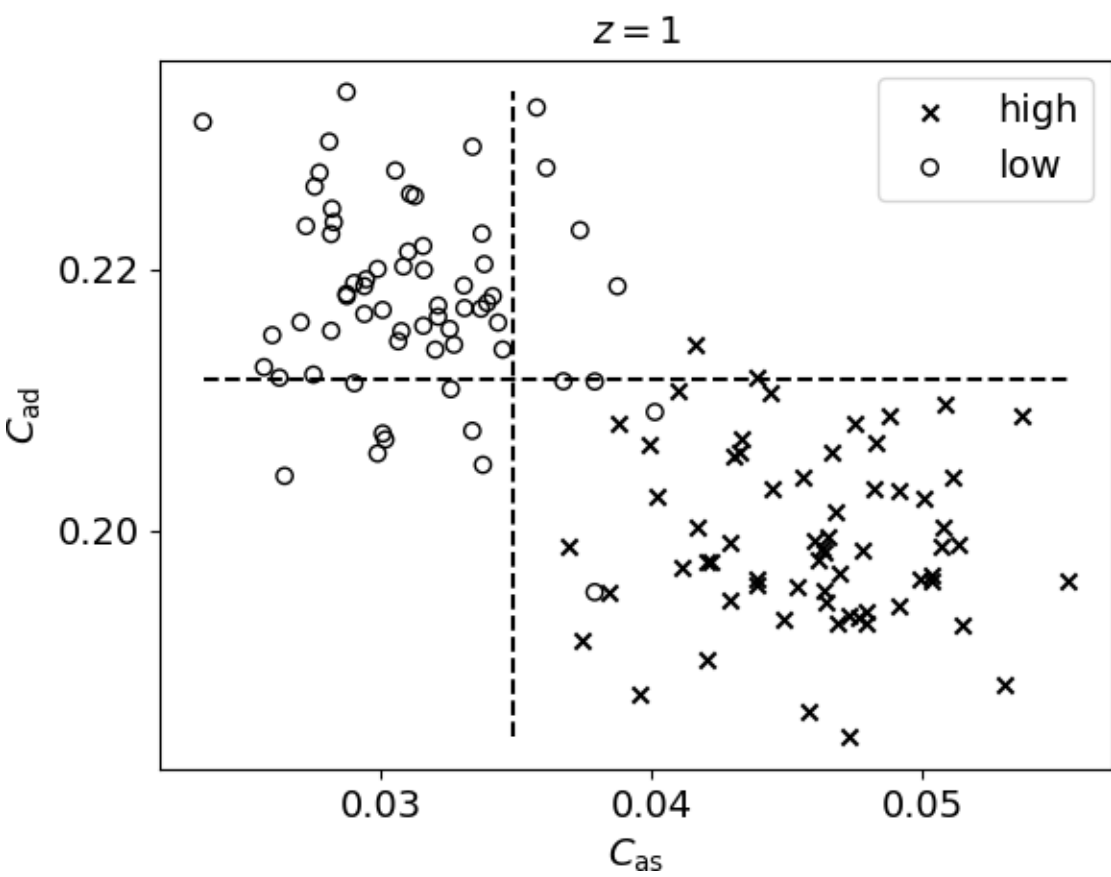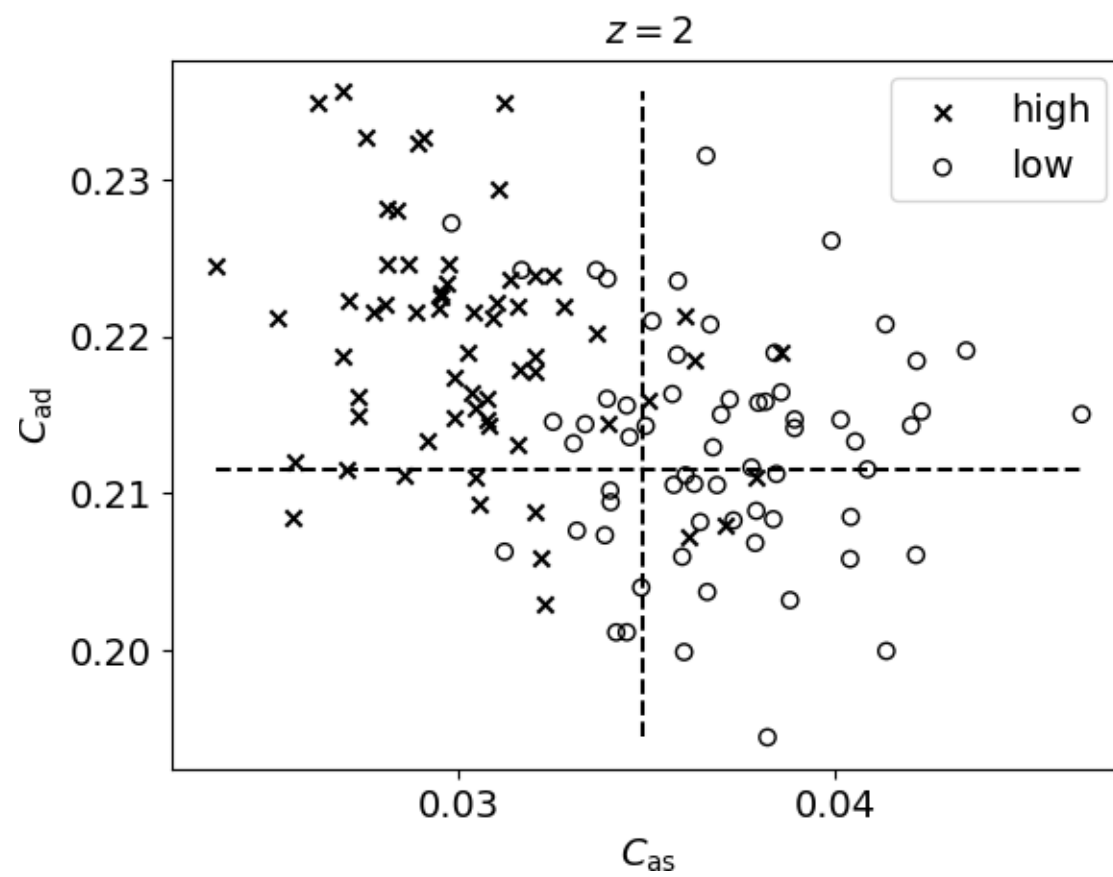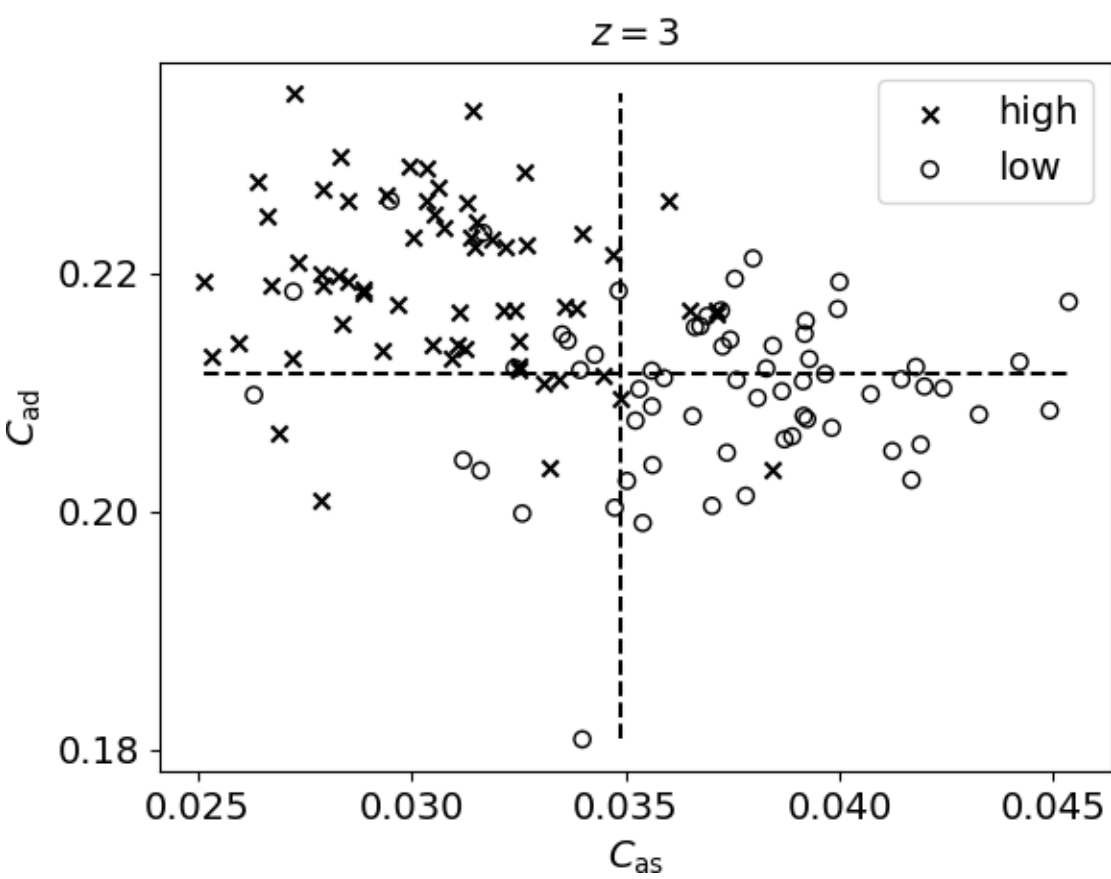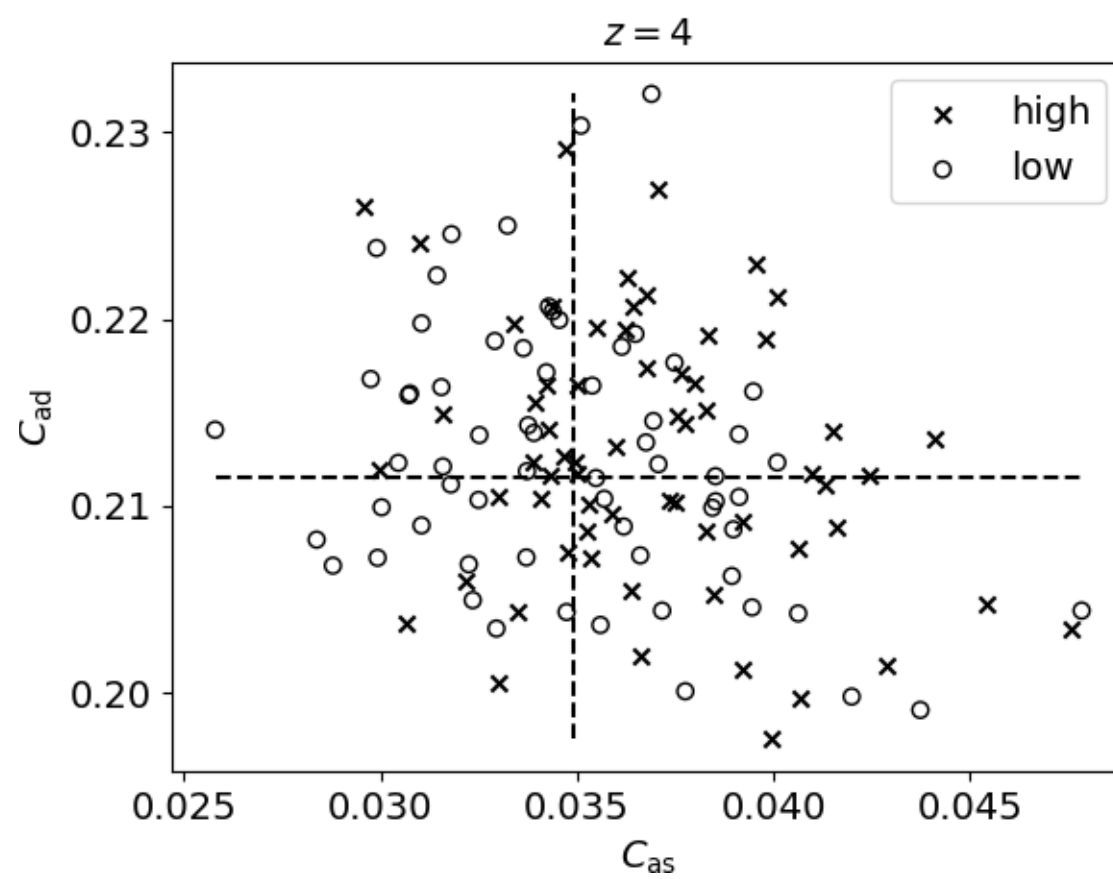

Supplement: S9 Fig — See Eqs (27) and (28) in Methods for definitions of Cas and Cad. Each data point (obtained from Model 2 for L = 64) indicates, for enhanced clarity, an average over 3 pairs of patterns. Crosses (open circles) represent correlations averaged over 3 most (least) frequent pairs, whose relative positions are determined by z in a latching sequence. Horizontal and vertical dashed lines indicate the average values of Cas and Cad over all patterns. At the first step (z = 1), latching occurs most frequently between highly correlated patterns, in agreement with previous studies on long-term memory. At the third step, the trend is reversed. (PDF) [file pcbi.1008809.s009.pdf]

(a)

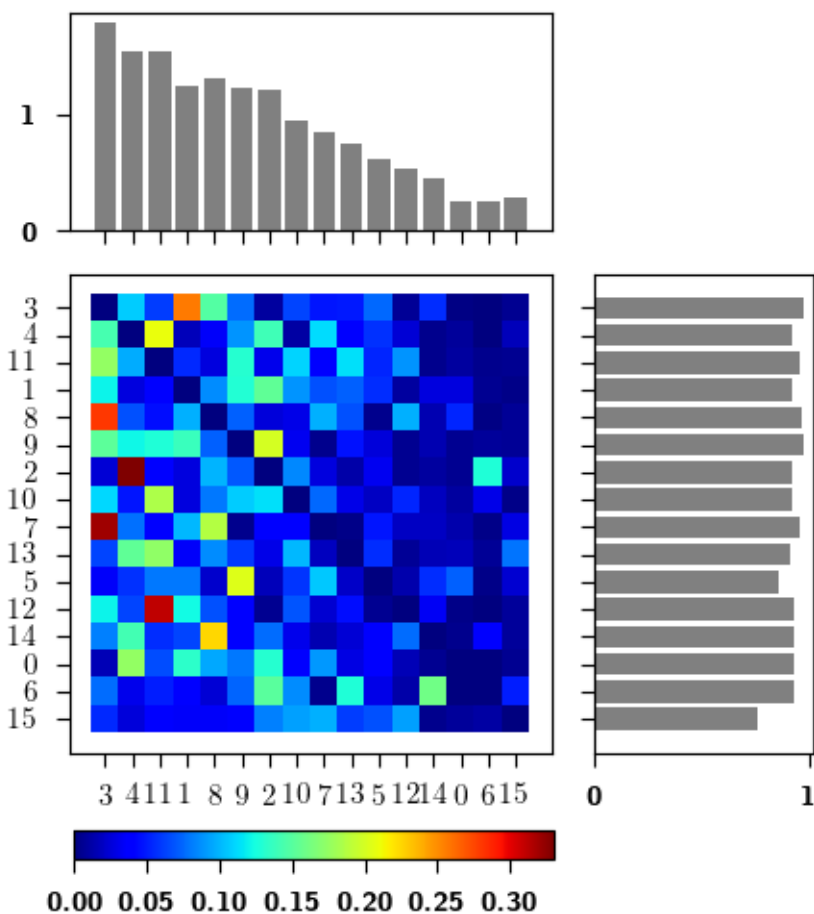

(b)

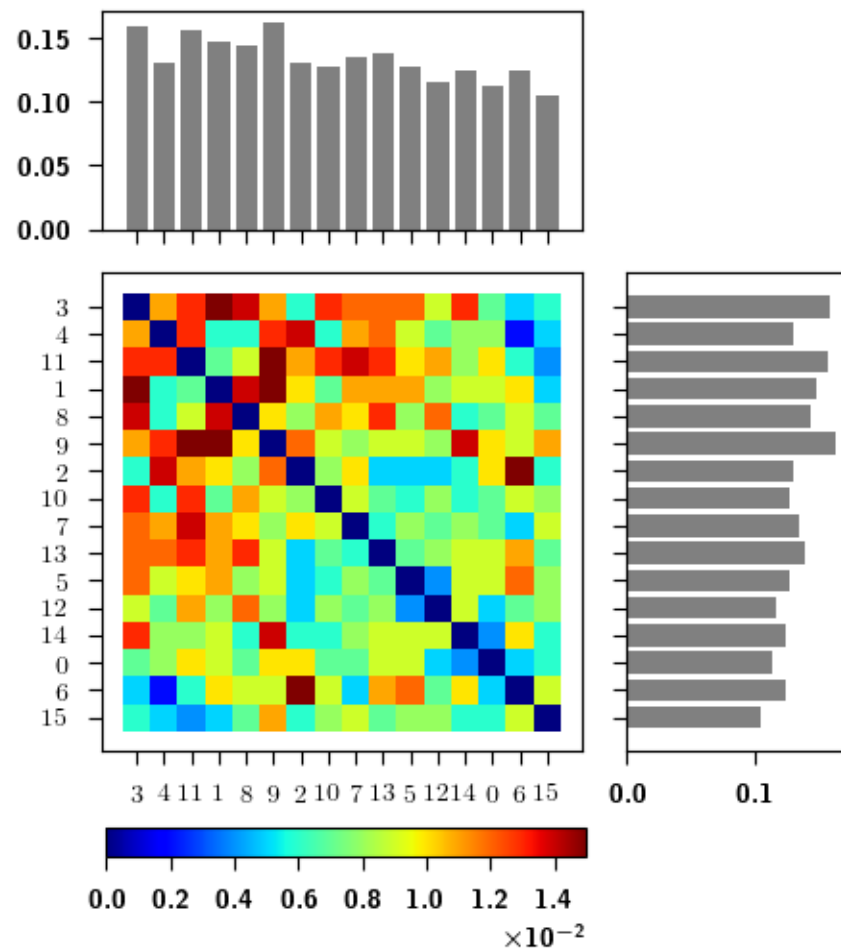

(c)

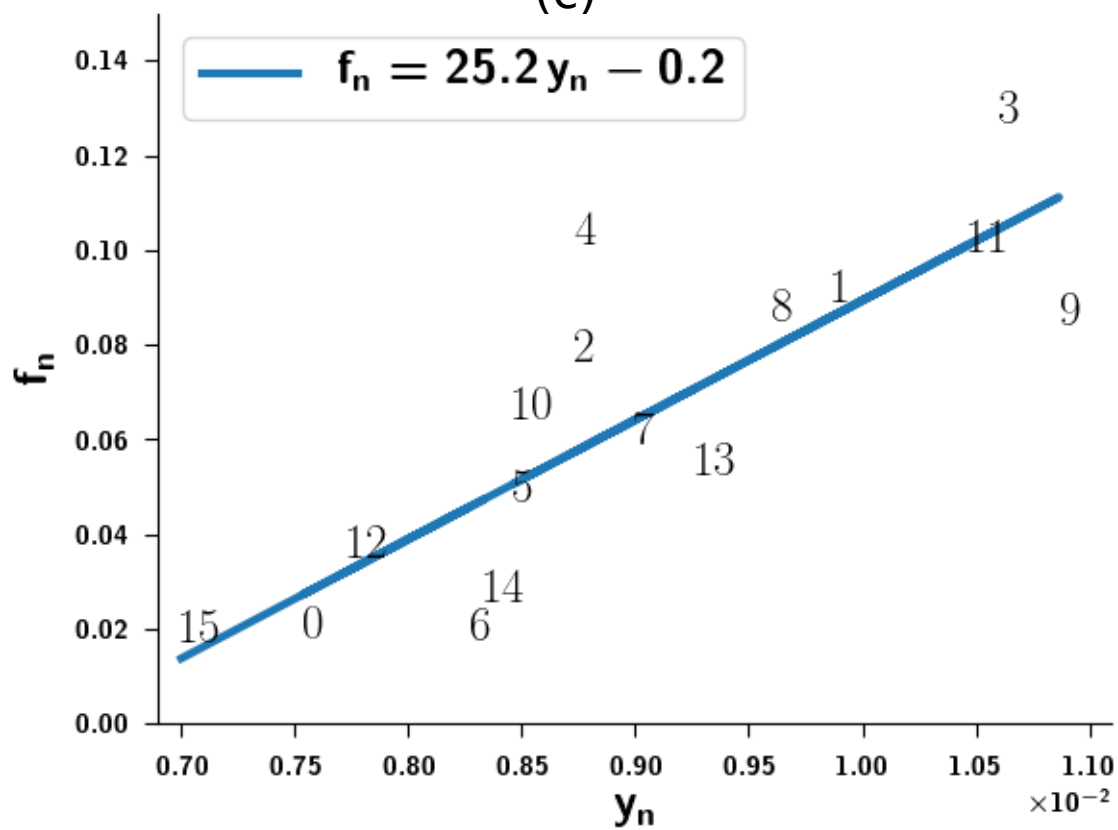

Supplement: S10 Fig — (a): Re-ordered transition matrix for p = 200 and L = 16 for one set of patterns, ordered according to the visit frequencies of each pattern in that data set. The matrix of transition probability has rows—where the network latches from, which in turn is just the probability of appearance of each pattern—that look roughly similar to the average row (with fluctuations), while the columns—where the network latches to—are very different from each other, from the heavy ones on the left to the light ones on the right. (b): Cas matrix (see Eq (27) in Methods for its definition), again ordered in the same way as in (a). The diagonal has been set to 0 artificially, in order for off-diagonal values to be more visible. (c): Mean correlation of each pattern in STM with all the others in STM, yn, versus its visit frequency fn for p = 200 and L = 16. Numbers indicate the pattern indices (16 of them). (PDF) [file pcbi.1008809.s010.pdf]

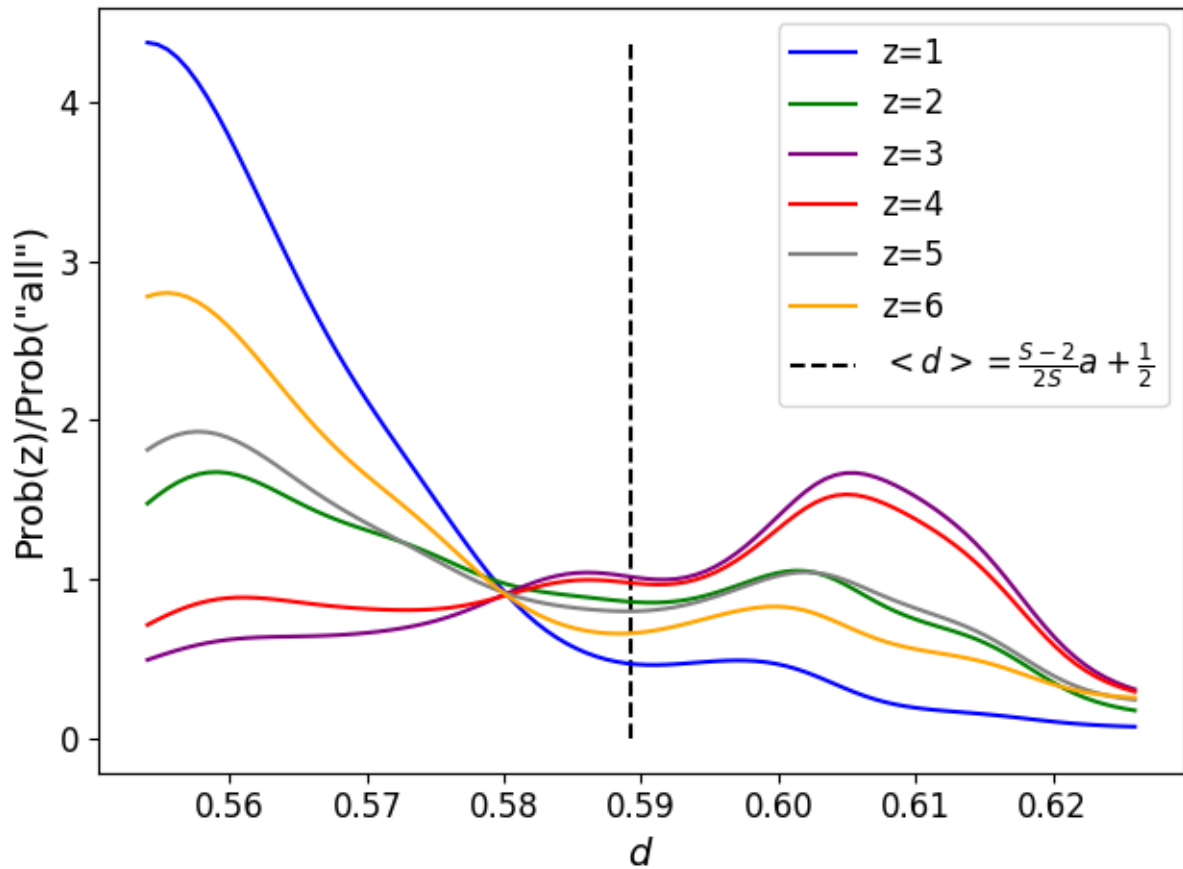

Supplement: S11 Fig — Probability density of d(μn, μn+z) (see Eq (22)) is divided by the probability density of d(μ, ν) for all possible pairs among L patterns in STM from simulating Model 2. From z = 1 to z = 6, we can see the quasi-periodic evolution of the probability density function. Parameters are w = 0.8, γA = 0.5, L = 16, Δθ = 0.3. (PDF) [file pcbi.1008809.s011.pdf]

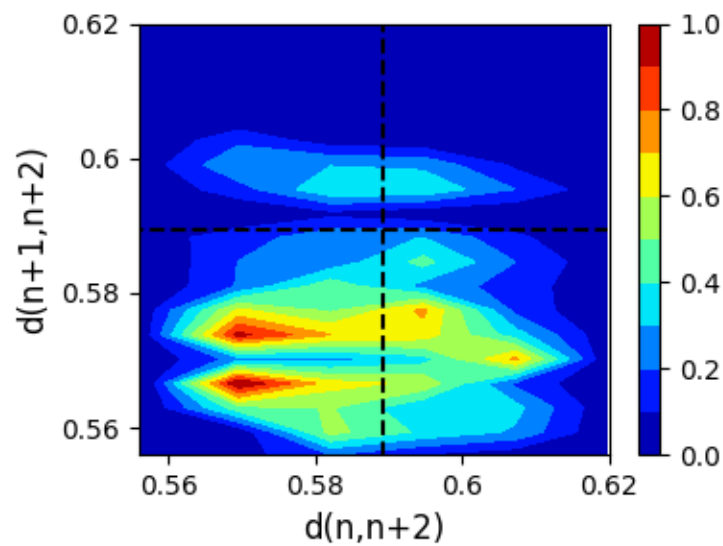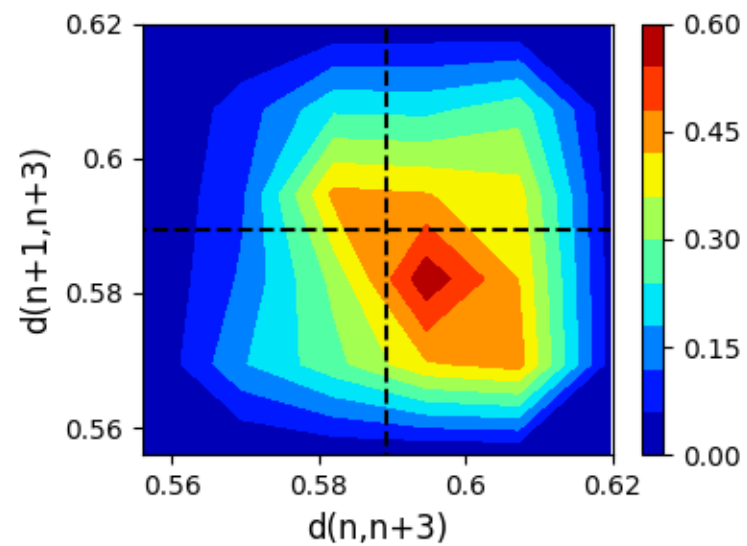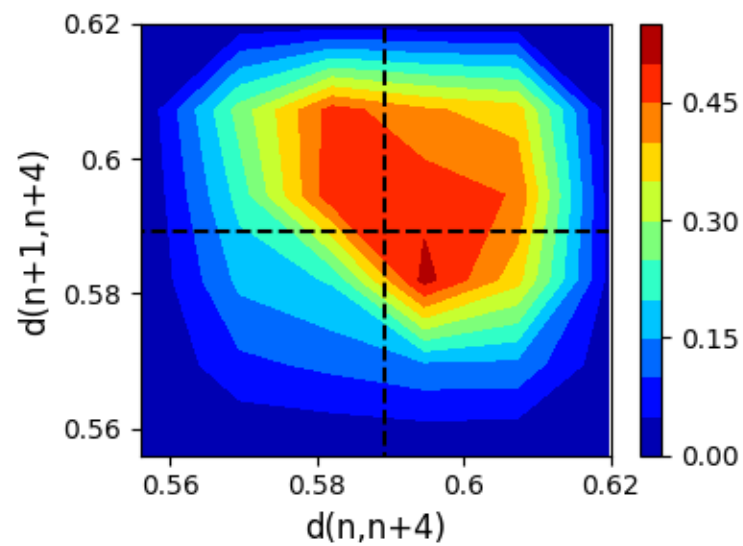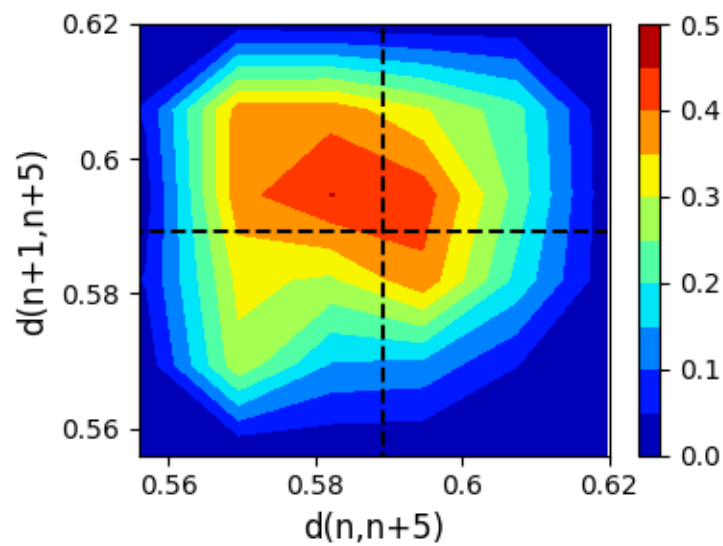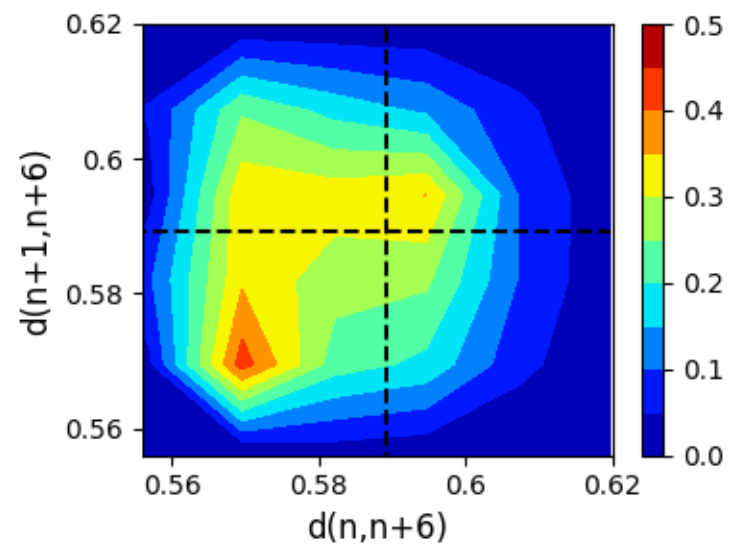

Supplement: S12 Fig — Colour indicates the visiting frequency. From the upper left panel to the lower right one, we can see that the brightest spot (most frequent visits) rotates counter-clockwise. Dashed black lines indicate the average value across all pairs in STM on the corresponding axis. Parameters are w = 0.8, γA = 0.5, L = 16, Δθ = 0.3. (PDF) [file pcbi.1008809.s012.pdf]

MI/Entropy

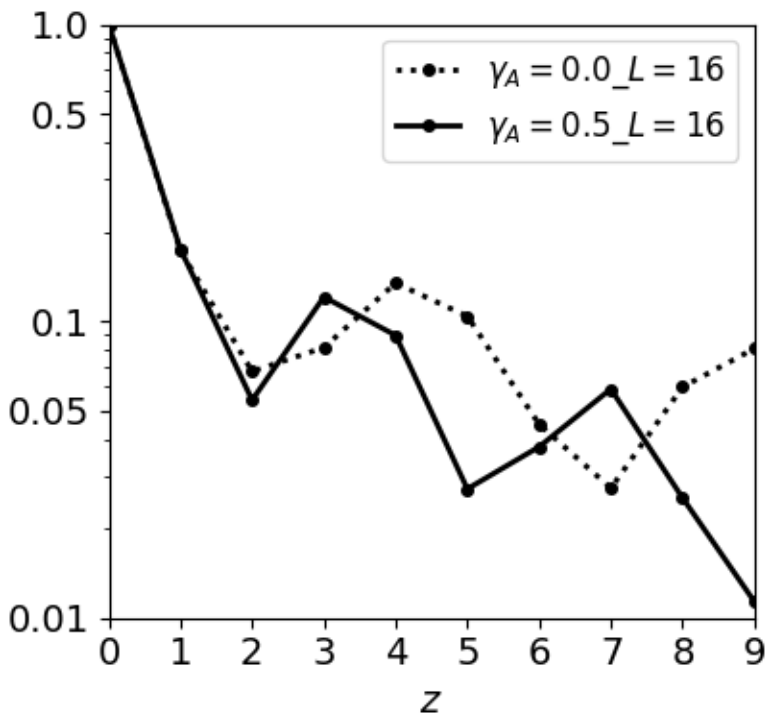

Supplement: S13 Fig — (PDF) [file pcbi.1008809.s013.pdf]
