# Supplementary material for: Latching dynamics as a basis for short-term recall
Source: PLoS Comput Biol. 2021 Sep 15;17(9):e1008809. doi: 10.1371/journal.pcbi.1008809 (PMC8476040; doi:10.1371/journal.pcbi.1008809)
Supplement: S1 Appendix — (PDF) [file pcbi.1008809.s014.pdf]

Appendix for "Latching dynamics as a basis for short-term recall": Deriving scaling law under the assumption of equal visits

The quantity M can be estimated under the assumption of equal visits to each of the patterns. Under such an assumption, the probability of going to a new item m times and to one already visited at the $m+1$-th time step is given by $1(1-1/(L-1))...(1-(m-1)/(L-1))m/(L-1)$ and this contributes to $M = m+1$. So taking a sum for m from 1 to $L-1$ of this probability times $m+1$ gives

$$M \equiv \sum_{m=0}^{L-1} \frac{m(m+1)}{L-1} \prod_{k=0}^{m-1} \left(1 - \frac{k}{L-1}\right). \tag{1}$$

One simple approximation of this expression for L large yields

$$M \simeq \sqrt{2(L-1)}\,\gamma\left(\frac{3}{2}, \frac{L-1}{2}\right) - e^{-\frac{L-1}{2}} + 1\,, \tag{2}$$

where γ is the *lower* incomplete Gamma function, which for $L \to \infty$ grows as a square root,

$$M \simeq \sqrt{(\pi/2)(L-1)} + 1. \tag{3}$$

One way to approximate this expression for L large is to assume $1 - \frac{k}{L-1} \simeq e^{-\frac{l}{L-1}}$, so that the product of the exponentials becomes the exponential of the sum, and one has

$$M \simeq \sum_{m=0}^{L-1} \frac{m(m+1)}{L-1} \exp\left(-\frac{m(m-1)}{2(L-1)}\right). \tag{4}$$

To further approximate the above sum with an integral, let $x = \frac{m}{\sqrt{L-1}}$, then we have

$$M \simeq \int_0^{\sqrt{L-1}} dx \left(\sqrt{L-1}x^2 + x\right) e^{-\frac{1}{2}\left(x^2 - \frac{x}{\sqrt{L-1}}\right)}. \tag{5}$$

Keeping only the first term in the exponent of the integral

$$\int_0^{\sqrt{L-1}} dx \left(\sqrt{L-1}x^2\right) e^{-\frac{x^2}{2}} = \sqrt{2(L-1)}\,\gamma\left(\frac{3}{2}, \frac{L-1}{2}\right) , \tag{6}$$

where

$$\gamma\left(\frac{3}{2}, \frac{L-1}{2}\right) = \int_0^{\frac{L-1}{2}} t^{\frac{3}{2}-1}\, e^{-t} dt \tag{7}$$

is the *lower* incomplete Gamma function, and

$$\int_0^{\sqrt{L-1}} dx\, x\, e^{-\frac{x^2}{2}} = -e^{-\frac{L-1}{2}} + 1. \tag{8}$$

An alternative expression for M is in terms of the *upper* incomplete Gamma function,

$$M \equiv \sum_{m=1}^{L-1} \frac{m(m+1)}{L-1} \frac{(L-1)!}{(L-1-m)!(L-1)^{m-1}} = \frac{e^{L-1}}{(L-1)^{L-1}} \Gamma(L, L-1). \tag{9}$$

To derive its asymptotic behaviour for large L, it is convenient to separate one term and write

$$M = 1 + \sum_{l=1}^{L-1} \frac{(L-1)!}{(L-1-l)!(L-1)^l}, \tag{10}$$

and then use Stirling's approximation for the factorial to evaluate the sum as half an indefinite integral for $-\infty < l < \infty$, which can be evaluated at its saddle point near $l = 1/2$, yielding again, to leading order, $M \simeq \sqrt{(\pi/2)(L-1)} + 1$.
