# Supplementary material for: Latching dynamics as a basis for short-term recall
Source: PLoS Comput Biol. 2021 Sep 15;17(9):e1008809. doi: 10.1371/journal.pcbi.1008809 (PMC8476040; doi:10.1371/journal.pcbi.1008809)
Supplement: S1 Table — (PDF) [file pcbi.1008809.s015.pdf]

Parameters of the network

Symbol	Meaning	Default value
N	number of Potts units	1000
S	number of states per unit	7
p	number of stored LTM patterns	200
a	sparsity of patterns	0.25
c_{m}	number of presynaptic units per unit	150
U	threshold common to all units	0.1
β	effective inverse temperature	11
τ_1	timescale for "fields" (r_i^k)	10
τ_2	timescale for adaptive thresholds (θ_i^k)	200
τ_{A}	timescale for fast inhibition (θ_i^A)	5
τ_{B}	timescale for slow inhibition (θ_i^B)	100000
γ_{A}	proportion of fast inhibition	0.5
w	self-reinforcement parameter	0.8
L	number of patterns in STM	16
$\Delta\theta$	the amount of decrease in adaptive threshold	0.3
λ	strength of heteroassociative connections relative to autoassociative ones	0.1
