# Supplementary material for: Latching dynamics as a basis for short-term recall
Source: PLoS Comput Biol. 2021 Sep 15;17(9):e1008809. doi: 10.1371/journal.pcbi.1008809 (PMC8476040; doi:10.1371/journal.pcbi.1008809)
Supplement: Attachment — Submitted filename: coverLetter_PCOMPBIOL-S-21-00280.pdf [file pcbi.1008809.s016.pdf]

Dear Editor,

Please find attached a new version of our manuscript, entitled “Latching dynamics as a basis for short-term recall”, which had received the tracking number PCOMPBIOL-D-21-00218 when first submitted.

We have worked hard on the manuscript to make it more readable following your comments on the 8th of March. We hope that the new version is clearer than the previous one, thanks to your helpful constructive suggestions.

Best wishes,
Kwang Il Ryom, Vezha Boboeva, Oleksandra Soltadkina, Alessandro Treves
