# Supplementary material for: Latching dynamics as a basis for short-term recall
Source: PLoS Comput Biol. 2021 Sep 15;17(9):e1008809. doi: 10.1371/journal.pcbi.1008809 (PMC8476040; doi:10.1371/journal.pcbi.1008809)
Supplement: Attachment — Submitted filename: answer_to_reviewers.pdf [file pcbi.1008809.s017.pdf]

Reviewer 1: Authors discuss retrieval capacity limitations using different measures and in different variation of Potts model for memory storage. In general, our knowledge of memory recall mechanisms in humans is very limited. Therefore, theoretical models, especially supported by experiments may help us constraint possible memory mechanisms. Current work presents some of the results in vague form that would better be sharpened before publication. For example, in lines 339-349 it appears that authors implicitly assume that the difference between long-term memory and short-term memory is in the way one measure performance, and not a separate mechanism. But this is so vague stated and buried in the rest of the text that it is not clear whether authors make this statement or not.

We thank the reviewer for the careful reading of our manuscript and apologize that, on this central point, our writing style has led to confusion: we do not assume that STM and LTM differ in how one measures performance. We rely on the wider, yet somewhat loose, usage of these terms as referring to memory expressed within and beyond a time scale of the order of seconds, at most minutes. Within STM, we argue that whether it is limited by very low capacity or not (relative to the potentially huge capacity of LTM) depends on how one measures performance. We have revised the critical passage in this way:

"Performance" therefore depends very differently on L, if recall is taken to be terminated by errors, i.e. by the erroneous recall of an item that is not in STM. Thus, while if ignoring errors the notion of STM *capacity* appears irrelevant (given the scaling behaviour of the various quantities discussed above), it becomes quite relevant if errors are considered to be critical in the task…

Authors are trying to advance the idea that capacity of recall is limited by items interference. Nevertheless, authors ignore the model that give estimated value of capacity close to one observed in experiments with humans. In contrast, they choose the model that has much larger expected capacity. The arguments to choose one specific model as the model for short-term memory are not convincing.

In all three simple models we consider, in fact, the capacity for recall, if terminated by errors, is limited by items interference. The utility of such simple models is that they indicate in each case the functional dependence of the limit on the parameters of the network. The numerical values of the limit would be real predictions if the parameters were firmly established, which they are not. The reviewer is obviously right that this point had to be better explained. We have added an extended explanation of the logic for considering the different models and then model 2 in detail. In particular, we have added the paragraph:

The effectiveness is measured, in Fig. 2c, by a quantity called M_{corr}, which is the number of recalled STM items until the network either repeats one of the already-visited items or retrieves one of the LTM items. We then consider the difference between this quantity and the value it would have without any differentiation between the L and the other items, $\Delta M_{corr} \equiv M_{corr}(\Delta x=0.3) - M_{corr}(\Delta x=0.0)$; this subtraction of the chance level quantifies the genuine effect of Δx. Here x represents w, ϑ and J for the 3 models, respectively. When we increase L, there are two main factors that affect Mcorr. The first one is the exploration by the trajectory, resembling that of a random walk, which increases Mcorr. Due to this effect M_{corr} should grow like $\sqrt{L}$ as a function of L (see Appendix) if there are no errors, i.e. recall of items that are not in short-term memory. The occurrence of errors is the second factor that affects M_{corr}, progressively more as L increases. When L is small, the first factor dominates and as a result, M_{corr} grows. Beyond a certain value of L, there is an avalanche of errors as there are many LTM patterns that are kicked as strongly as those in STM. This avalanche of errors causes the sudden drop of ΔM_{corr} seen for Model 2 and 3a in Fig. 2c.

Nevertheless, results of experiments are roughly consistent with simulations. Therefore, it is possible that the model describes some aspects of recall. In my opinion the manuscript should be revised to make points made clear.

Minor points.

Lns 112., 115. The same letter theta used for different variables which is confusing.

Thank you for indicating the potential confusion. It is true that this notation might cause confusions, but we would rather keep it as it is for consistency with previous publications. Several previous papers consistently use this notation, so it might cause further confusion if we change it here.

Ln. 129 “step towards plausibility” probably means towards realistic network

Yes, we corrected. “step towards plausibility” → “step towards a realistic network”.

Lns 132-145. The statements may be more precise. (ln. 133 “perfectly retrieved”, ln. 136 “after the retrieval” what means retrieval? Perfect, above threshold on overlap?).

We changed some words to be more precise as suggested by the reviewer.
“measures the retrieval of each pattern” → “measures the distance between the network state and each pattern”.
“When $\m^\\mu=1$, pattern μ is perfectly retrieved.” → “The overlap $m^\\mu$ is normalised in such a way that it takes the value of 1 when the network state is fully aligned with one pattern.”

(ln. 137 “Above phase transition” - is there a single parameter defining phase space?)

In Fig.1b, we took the local feedback *w* as the control parameter for the phase transition just to show an illustrative example. Of course, other parameters of the network can cause phase transitions. To make things clear, we added the following sentence:
“The Potts network undergoes a phase transition by changing one of the network parameters (e.g., the local feedback *w* in Fig. 1b).”

(ln. 143. “but otherwise free” - ???)

The Potts network shows a spontaneous latching sequence, which resembles a random walk in pattern space. When the network is to serve a STM function, we adjust one relevant variable to restrict the latching sequence to the subset of patterns that are supposed to be in STM. Except for this restriction, there is no further restriction on the latching sequence resembling the random walk. This was the meaning of “otherwise free”. We changed the sentence as follows:
“As the network hops from memory to memory, it can simulate free recall, if latches are concentrated onto STM items, but otherwise free.” → “As the network hops from memory to memory, it can simulate free recall, if latches are concentrated onto STM items, but otherwise free, i.e., not coerced by external agents.”

Ln 214-215. “latching dynamics are effectively constrained to the L items, but only up to a given value of L” – meaning is not clear

We added several sentences to make it clear. Namely, we added after the above sentence the text already mentioned above:

The effectiveness is measured, in Fig 2c, by a quantity called M_{corr}, which is the number of recalled STM items until the network either repeats one of the already-visited items or retrieves one of the LTM items. We then consider the difference between this quantity and the value it would have without any differentiation between the L and the other items, $\Delta M_{corr} \equiv M_{corr}(\Delta x=0.3) - M_{corr}(\Delta x=0.0)$; this subtraction of the chance level quantifies the genuine effect of Δx. Here x represents *w*, ϑ and J for the 3 models, respectively. When we increase L, there are two main factors that affect M_{corr}. The first one is the exploration by the trajectory, resembling that of a random walk, which increases M_{corr}. Due to this effect M_{corr} should grow like $\sqrt{L}$ as a function of L (see Appendix) if there are no errors, i.e. recall of items that are not in short-term memory. The occurrence of errors is the second factor that affects M_{corr}, progressively more as L increases. When L is small, the first factor dominates and as a result, M_{corr} grows. Beyond a certain value of L, there is an avalanche of errors as there are many LTM patterns that are kicked as strongly as those in STM. This avalanche of errors causes the sudden drop of ΔM_{corr} seen for Model 2 and 3a in Fig 2c.

Ln 241. "ita** regime of operation, such that **the its** ability to spontaneously recall"**

We changed: "ita" → "its"; "the its ability" → "its ability".

Section 3. It is not clear why one would like to have more latches that the number of stored memories. It is probably more important how many unique memories are recalled (latched).

Definitely in many tasks, it is important how many unique memories are recalled, and so it is in evaluating the recall of "performance" of the Potts network. We have also measured the number of latches in order to assess how effective each model is in restricting latching dynamics into a subset of *L* patterns out of all *p* patterns. Our model comparison goes in this way. We prepare the homogeneous network (without any boost for STM functionality) in the no-latching phase. Then we give an extra "kick" to L patterns, to temporarily hold them in STM, by adjusting, depending on the model, a specific parameter of the network. Before analysing the recall performance in each model, we evaluate its latching propensity as a function of the strength of the kick.

We added three sentences to make things clearer:

At line 264 (line number is from the new manuscript), "The Potts network becomes able to serve an STM functions once it undergoes a phase transition from the no-latching to the finite-latching phase. For this reason, we are also interested in the propensity to latch expressed by each of our models."

At line 278, "Note that the representation of objects has been found to be "enhanced" in working memory tasks [24], likely with higher neural activity in the participating units [25], broadly consistent with Model 2."

Lns 339-349. The meaning of these two paragraphs are not clear. Do authors suggest that STS is not a brain mechanism but an artifact of quantification method? There are some modeling works in psychology that does not require STS per se to describe free and serial recall (e.g. doi:10.1037/0033-295X.114.3.539). But the rest of text is inconsistent with this statement. So, I guess, authors can make their statement more precise here.

Thank you for indicating the potential misunderstanding in our statement. We did not try to state that STM is an artifact of quantification method. We now better clarify this point by expanding the following sentence at line 396:

"In summary, we have shown that whether we get scaling or saturation in STM performance depends on the specific metric we use to measure it, both in the Potts network, endowed with an STM mechanism and in our experiment."

Ln 368-369 "In this way we measure the memory capacity for serial recall by computing the Area Under the Curve (Fig. 6b)." – Could authors be more precise what exactly was done and why AUC appears here?

Thank you for your point. We changed:
"In this way we measure the memory capacity for serial recall by computing the Area Under the Curve (Fig 6b)" → "Fig 6B shows the memory capacity for serial recall in this task (see Methods for how we measure memory capacity)".

We added one paragraph in the subsection of "Serial recall" of the section "Methods", where we explained how one can measure memory capacity in the serial recall task.
The added paragraph reads,
"In order to measure memory capacity in this serial recall task, we first plot the proportion of correct trials as a function of *L*, either for each participant in Fig 6b or for the pooled data across all participants in Fig 7a. Although the minimum *L* we used was 3, we added two "data points" by hand to the proportion *P(L)*, setting it to 1 (i.e., a putative 100% for *L*=1 and *L*=2). We then compute the memory capacity as the simple sum,
\begin{equation*}
C = \sum_{*L*=1}^{L_{max}} *P(L)*,
\end{equation*}
where L_{max} is the maximum value of *L* used in the experiment. This measure is usually referred to as Area Under the Curve or AUC [51]".

Ln 387 "such sequences as Potts-compatible" – that may be too strong statement, since one may be able to implement in the future Potts model that can recall arbitrary sequence. Reserving this strong term then would lead to confusion. This is not critical, for current presentation though.

Thank you for this comment. Yes, we introduced necessary changes.
We removed the sentence "Due to this, we refer to stimulus sets excluding such sequences as Potts-compatible".

In the Methods, we change,
"We call sequences without any subsequence of the form ABA and AA *Potts-compatible*. In this way we prepare a set of 80 Potts-compatible sequences for a given value of *L*" → "Sequences without subsequences of the form ABA and AA are favored by the Potts network. So, we prepare a set of 80 sequences that do not include any subsequence of the form ABA and AA, for a given value of *L*".

Ln 409-410 "These instructions are congruous, as they reproduce latching sequences emerging without any heteroassociative instructions" – it is not clear what is mathematically meant by instructions.

We try to make things clearer:
"To see this effect, we obtain some intrinsic latching sequences by running simulations with $\lambda = 0$; from these latching sequences, we generate a set of instructions for the serial order. These

instructions are *congruous*, inasmuch as they reproduce latching sequences emerging without any heteroassociative instructions."

The mathematical meaning of "instructions" is given by Eq. (24) in Methods. Intuitively, these "instructions" tell the Potts model about the serial order of items in STM. The word "instruction" and its meaning are explained on the line 450 and below.

Ln 415 "can serve short-term memory (e.g., free recall)" – meaning is not clear

We try to make it clearer by changing:
"e.g., free recall" → "e.g., they can be utilised for free recall"

Fig. 7 Blue dotted curve (a) 0.6+0.4+0.1 << UAC 3 in (c) "(c) Area Under the Curve (AUC) computed from the curves of (a), intended as a measure of overall performance."

This was addressed by adding the above paragraph to the subsection "Serial recall" in the section "Methods". The inconsistency between panel (a) and (c) that you mentioned comes from the fact that we added by hand two fictitious data points: the proportion is 1.0 for L=1 and L=2, although trials always started with L=3 (what is now clarified in Methods).

And the legend was changed:
"(c) Area Under the Curve (AUC) computed from the curves of (a), intended as a measure of overall performance" → "(c) Memory capacity computed from the curves of (a) (see Methods)"

Ln 431. Cad is referred (Fig S10) before it is defined.
Ln 433 Cas is referred (Fig S11) before it is defined.

Thank you for this point. We added a reference to the definitions of Cas and Cad for this problem.

Reviewer #2: The authors present a model of free and serial recall, which embodies the idea of short-term memory as the result of "priming" a sub-set of items in the long-term memory store. The long-term memory store is implemented by using a Potts model, and three different mechanisms for priming items are introduced and studied. The model is able to reproduce experimental observations, notably the "square root law", and provide interesting insights into possible mechanism(s) responsible for the limited capacity of short-term memory.
Recent work by Tsodyks and collaborators has shown that a random walk in "memory space" can account for subjects' short-term memory performances to an amazingly quantitative detail. This work follows up on that thread and provides a more "microscopic" interpretation of the memory random walk. Importantly, in my opinion, it shows that the hypothesized dynamics can indeed occur in a distributed memory system; and strongly suggests that capacity limits are ultimately due to the distributed nature of the long-term store.
The results reported appear correct, as far as I can check. The paper is very well written and the figures are well chosen and informative. I have no comments.

We thank the reviewer for the appreciation.

Reviewer 3: The paper by Il Ryom et al is a great contribution for understanding the memory of sequences and short term memory of multiple objects. The authors employ their previously developed Potts network model of cortical memory processing and consider modifications of it

(e.g. adaptation, synaptic plasticity etc) that allow a study of how short term memory of multiple objects as well as sequences can be formed from the stored long term memory patterns. In addition to this, they offer experimental data that allow understanding how each of these different modifications contribute to the errors (or regularities in the errors) that appear in the data. I think this work is important, as theoretical models for memory of sequences and short term memory, the capacity of such memories, and type of errors that they lead to have been paid much less attention to. This is particularly the case when compared to working memory models of e.g. objects, or models of long term memory. When it comes to sequences processing models that have been studied, more or less all rely on the the standard way of storing and retrieving sequences follow the seminal work of Sompolinksy & Kanter PRL 1986 where hetro-associative connectivities are employed, without much studying how the presence of long term stored memories influence errors in retrieval. The current paper takes this further by considering how other mechanisms such as adaptation and intrinsic latching (the ability of the network to generate quasi-random sequences of long term stored patterns) interact with this heteroassociative connectivity. The paper also provides a mechanistic model of the sqrt(L) law in free recall which is also quite novel. So, in principle, the paper is a suitable contribution to PLoS Comp Biol, though I think before publication, as I describe below, a number of changes should be done and a number of issues explained.

1. The first point that I think needs to be addressed by the author is how they pitch their work. The authors start by discussing their aim of explaining the low capacity of short term memory. For this, they cite a number of experimental results. The problem that I found confusing was that short term memory is one of those words that mean different things to different people. In the introduction and throughout the paper, the authors mention a range of experimental results from change detection experiments in visual working memory tasks to free recall tasks, some making quantitative observations, some qualitative, although all point to the direction of limited capacity (however defined in that particular task) of memory in those tasks. The model the authors describe, however, as far as I understand, is mainly about a system that generates a sequence of a certain number of objects in a given order, or quasi-randomly (e.g. in a free recall task). How, for instance, this relate to change detection memory tasks is not immediately clear.

I think the paper would benefit from a more focused introduction, and perhaps a section reviewing what in different and specific experimental setups people define as short term memory, what they use to measure capacity, and how the model described here relate to each. This I believe would be extremely useful for the audience of PLoS Comp Biol who may not all know all the different ways people define short term memory in experiments and how these different ways relate to each other.

The reviewer is right, of course, that different people refer with STM to different phenomena, most of which have or are presumed to have something in common, but which also differ in several respect. We have considered considerably expanding the introduction to discus this complexity, but then felt that it was distracting from the specific contribution that can be offered by the Potts network endowed with an STM model. Therefore, we focused on explaining it more clearly, and its relation to the simple experiments we have run ourselves, which serve as examples of the range of phenomena the model may be relevant to. Moreover, we refer to authoritative papers, and in particular to Oberauer et al, 2018, for insider reviews of the complexity of STM and WM phenomenology (particularly in humans).

2. In estimating L_c, the authors use the criterion P_L= 1-1/e combined with Eq. 14-17. where does this criterion come from?

The criterion is arbitrary, as the reviewer has likely guessed, but it is expressed in a convenient manner, because in the models we consider P_L approaches 1.0 as L grows, in the form 1-(1-k)^L (except for model 3B), so the criterion is expressed as a simple condition on k. Alternative criteria, for example P_L=0.5, would lead to equivalent but somewhat more complicated expressions.

3. In sec 4.2, the authors define g(L) as the given number of consecutive latches and then set this to 4 log-2(L)-2 to "establish a reasonable comparison with the results in [12]". Can you elaborate where this comes from?

As explained in the second sentence of section 4.2, the time duration given for recall in [12] starts with 90 seconds for L=4 and it is increased by 90 seconds when L is doubled. Mathematically, $t = 90*\log_2(L)$ - 90 sec., for L=4, 8, 16, 32, etc. We couldn't establish a more precise correspondence between "free recall" by our model and free recall in [12]. So, we kept only the functional dependence on L, as a log function.

4. Section 6. I think it would be useful if the authors explicitly give the expression for the mutual information between patterns. This can be defined in a number of ways, and it is only from the context that one can infer what the authors mean by it. Same section, z is defined as the relative separation. Relative to what?

Thank you for the comment. We added the explicit formula and an explanation of z in the Methods.

5. Again in section 6, it seems to me that the authors make a number of guesses that are not clearly stated.
For instance, in line 442, they state where the second order peak would be located but don't say how they come to this conclusion, or how what zeta is twice the z-value of the first peak.
I also think it is better to refer to the peaks as first and second peaks instead of first order and second order peaks.

Thank you. We have changed the word, "second order peak" → "second peak" .
We have also added one more plot in the supplementary figure (S13 Fig), indicating the positions of the second peak. Because of limited sampling, it is difficult to get reliable data for even larger values of z, the figure indicates that indeed the dependence on z resembles a damped oscillation, with the second peak at about twice the value of the first, e.g., for γ_A=1.0.

6. Last sentence of section 6 "This is consistent ...". I think the damped oscillation results in this section are quite interesting and it would be appreciated if the authors elaborate on the consistency with the results of sec 13 and/or discuss other possible ways experiments can support or have supported this phenomenon.

Thank you for the appreciation. While hoping to be able to identify relevant experimental measures, we think the data points to Fig. 8 make the damped oscillation notion much more convincing.

7. Regarding the damped oscillatory behaviour of sec 6: could this be simply understood by the fact that there is a competition between adaptation (making a more correlated patterns less likely to follow) and correlations (making a more correlated pattern to be follow)? If so, is it not possible to write down effective dynamics that describe the phenomena based on these two?

This is indeed a most interesting suggestion, that we leave however for further study.

Throughout the paper the authors repeatedly mention the papers from Misha Tsodyks' group (refs 12 and 25). I think it would be useful if the authors give a name to this model, instead of using they and their together with the refs.

The model by Sandro Romani, Misah Tsodyks and colleagues revises the notion of probabilistic search proposed in their "SAM" theory by Raaijmakers and Shiffrin [28], but also it resolves the issue of how the search is terminated with deterministic dynamics. Henceforth, for simplicity we will refer to it as the SAM++ Model. We have modified accordingly several passages in the manuscript.

line 87: which model patches of cortex--> each of which representing the state of a single patch of the cortex

We did make the change.

line 90: one quiet state: perhaps it should be said that this a state where no pattern is retrieved, e.g. a background firing state, not necessarily a silent or no-firing state.

Yes, we have changed it.

line 106: current--> input

Changed.

line 111: effective inverse temperature is a physics jargon. I would say that beta measure the level of noise in the system.

Yes, we have changed it.

line 141: revise the sentence. I cannot parse it.

We have clarified the sentence, we believe, by adding a coda.

As the network hops from memory to memory, it can simulate free recall, if latches are concentrated onto STM items, but otherwise free, i.e., not coerced by external agents.

line 152-155: long and difficult sentence to parse. it would perhaps be easier to say "are strengthened by increasing the value of some pre-existing parameters e.g. .... This increase effectively brings ...". Also I am not sure being items across a "network phase transition" means.

We have tried to clarify and make things easier to parse. The modified sentence reads, "The core idea is that a few memory items, or sequences of items, are strengthened by increasing the value of some pre-existing parameter (Fig 2a). The increase, which cannot be presumed to be precisely determined, should be in any case moderate, to effectively bring only those items across a network phase transition, into a phase in which they or their sequences are held in short-term memory, effectively separate from the ocean of all items and all possible sequences in long-term memory (Fig 2b). "

line 241: its--> its

Yes, corrected.

lines 491-494: "We have ..." long sentence, hard to parse and I think incomplete.

We have broken the long sentence into two:
“We have observed that, at least in the Potts network, if the heteroassociations, which amounts to specific instructions, dominate the dynamics, the random character is lost. With it we lose the entire latching dynamics -- which cannot be harnessed to just passively follow instructions.”

line 459: wouldn't it better to use discrete instead of digital? digital is often taken to be equivalent to binary though it is a wrong take.

Good idea, thanks. Amended.

Line 504: “paradigms that involve multiple subset”. Please elaborate what this means.

We have added here a sentence to elaborate, by offering an example.
“For example, in analyses of the Phonological Output Buffer (POB) the hypothesis has been considered of mutiple POBs, one holding simple phonemes, one function words, one numerals, etc., conceptually as sort of separate drawers, or *mini-stores* [41]”

line 503: what do you mean by "the time course of the "boost"."

We have now explained it better by changing:
“on the time course of the “boost” in the Potts model” → “on the time course of the “boost” that models STM in the Potts network”
